# Nociceptin Receptor Antagonism Modulates Electrophysiological Markers of Reward Learning

**DOI:** 10.1093/ijnp/pyad031

**Published:** 2023-06-20

**Authors:** Ann M Iturra-Mena, Brian D Kangas, Diego A Pizzagalli

**Affiliations:** Harvard Medical School, McLean Hospital, Belmont, Massachusetts, USA; Department of Psychiatry, Columbia University, New York, New York, USA; Harvard Medical School, McLean Hospital, Belmont, Massachusetts, USA; Harvard Medical School, McLean Hospital, Belmont, Massachusetts, USA

**Significance Statement**

Using in vivo electrophysiology and a reward-learning task reverse-translated from humans, this study tested the hypothesis that a nociceptin (NOP) receptor antagonist (J-113397) would potentiate behavioral and electrophysiological markers of reward learning in rats. Relative to vehicle, the NOP antagonist modulated electrophysiological markers of reward processing but did not affect response bias toward a more frequently rewarded stimulus. This proof-of-concept study provides initial insights into the effects of NOP receptor antagonism on reward learning, which are consistent with previous findings suggesting that such mechanism is a promising antidepressant target.

## INTRODUCTION

Anhedonia, the reduced reactivity to rewards, is a cardinal phenotype of major depressive disorder (MDD) (Pizzagalli, 2022). Nociceptin/orphanin FQ peptide (NOP) and its receptor (NOPR) have been implicated in various domains affected by MDD (e.g., learning, stress regulation, hedonic responses) (Gavioli et al., 2021). Particularly relevant, NOPR activation inhibits dopamine, leading to reduced motivated/hedonic behaviors (Gavioli et al., 2021), whereas NOPR blockade has antidepressant-like effects in rodents (Redrobe et al., 2002; Rizzi et al., 2007). While valuable, rodent assays probing anhedonic behaviors are typically very different from human tasks, which hinders translation. To fill this gap, we developed functionally identical versions of the probabilistic reward task (PRT) to objectively quantify reward responsiveness in humans and laboratory animals (Pizzagalli et al., 2005; Kangas et al., 2020). By unevenly distributing rewards between 2 difficult-to-discriminate stimuli, the task assesses the subject’s ability to develop a response bias (i.e., preference for the stimulus more frequently rewarded). Critically, individuals with MDD, and specifically those with anhedonia, show a reduced response bias (Pizzagalli, 2022).

Recently, we recorded local field potentials (LFPs) from 2 key brain reward nodes—the anterior cingulate cortex (ACC) and nucleus accumbens (NAc)—in rats performing the PRT (Iturra-Mena et al., 2023). We reported that 3 electrophysiological markers linked to reward processing in humans could be reliably detected in rats: an event-related potential (ERP) deflection 250–500 milliseconds after reward, as well as power increase in delta (1–5 Hz) and alpha/beta band (9–17 Hz) for rewarded trials. Consistent with human findings implicating delta and beta oscillations in reward prediction errors (i.e., when outcomes are better than expected) (HajiHosseini et al., 2012; Cavanagh, 2015; Marco-Pallares et al., 2015), delta (200–600 milliseconds) and beta (100–200 milliseconds) power in the ACC and NAc were largest after reward feedback, particularly for the less frequently rewarded stimulus (i.e., the largest reward prediction error). Building on these findings, we tested whether a single dose of the NOP antagonist J-113397 would potentiate response bias and electrophysiological markers of reward learning.

## METHODS

### Procedures

We conducted secondary analyses of Iturra-Mena et al. (2023), focusing on pharmacological effects. Eleven rats (5 females) were trained on a rodent touchscreen PRT (Kangas et al., 2020), with 100 trials divided across 3 blocks (block 1 and 2: n = 33, block 3: n = 34). Following PRT training, we implanted each animal with electrodes in the ACC/Cg2 (AP: +1.2, ML: +0.8, DV: −3.0) and NAc (AP: +1.2, ML: +0.8, DV: −7.0) for LFP recordings. In each testing/recording session, subjects were injected either with vehicle or J-113397 (10 mg/kg) 15 minutes before PRT testing. In this initial, proof-of-concept study, only 1 dose (10 mg/kg) was selected after (Genovese and Dobre, 2017), which demonstrated that 7.5-mg/kg and 20-mg/kg doses safely mitigated stress-related behavioral effects without causing behavioral disruption. Thus, we deemed a 10-mg/kg dose safe/suitable. ERP analyses and wavelet frequency-decomposition were performed as described (Iturra-Mena et al., 2023). Electrophysiological variables were computed time-locked to reward vs nonrewarded stimuli separately for the stimulus associated with more (rich) vs less (lean) frequent rewards. The current research was approved by the McLean Hospital’s Institutional Animal Care and Use Committee.

### Statistical Analysis

For drug-vehicle comparisons, we performed 3-way ANOVA using feedback-locked amplitude/power values on correct trials, entering reward feedback (rewarded/nonrewarded), stimulus type (lean/rich), and treatment (J-113397/vehicle) as repeated measures. For significant triple interactions, follow-up 2-way ANOVAs were performed to disentangle effects, followed by Šídák’s test for multiple comparisons. For response bias, a 2-way repeated-measures ANOVA with treatment and block (1, 2, 3) as factors was conducted.

## RESULTS

### Response Bias

**Figure 1.**
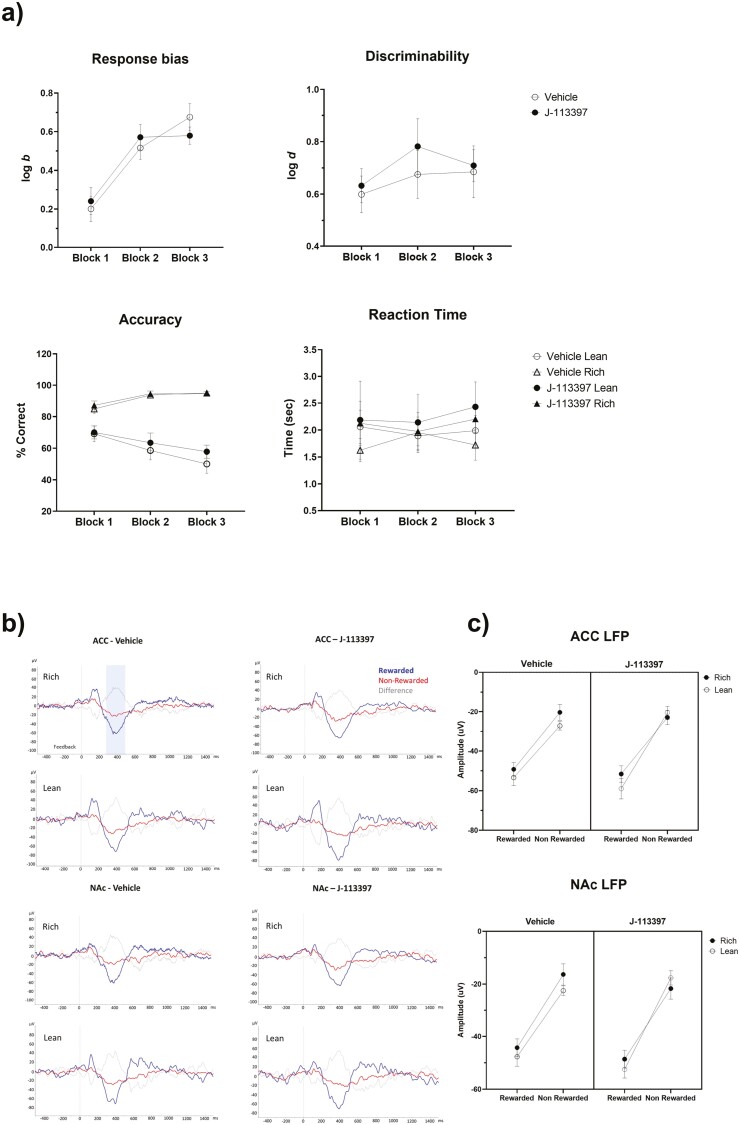
Effect of the NOP antagonist J-113397 (10 mg/kg) on behavior and the feedback-related positivity. (A) Behavioral performance for all conditions across 3 blocks. Upper panels: Response bias (left) and discriminability (right) calculated with log b and log d, respectively (for calculation procedure, see Iturra-Mena et al., 2023; Kangas et al., 2020). Bottom panels: accuracy calculated as the percentage of correct responses (left) and reaction time (right) measured as time to make a response (seconds). The PRT elicited the intended preference for the stimulus paired with more frequent reward (log b), without fluctuations in task difficulty (log d) or reaction time throughout the task for all conditions. Contrary to our hypotheses, J-113397 did not potentiate the animal’s preference for the more frequently rewarded stimulus (log b). (B) Grand average of the feedback-locked ERP for rewarded (blue), non-rewarded (red) trials, and the difference between them (gray) separated by stimulus type. A feedback-related positivity was observed as a negative deflection at 250–500 milliseconds after feedback in the ACC and NAc local field potentials. (C) Amplitude values for the ERP 250–500 milliseconds after feedback in ACC and NAc for all correct rewarded and nonrewarded trials separated by stimulus type—lean (white circle) and rich (black circle). A significant treatment × reward feedback × stimulus type interaction emerged for the ACC and post hoc tests further clarified that the interaction was driven by more positive deflection to nonrewarded lean trials for J-113397 than vehicle, albeit at a trend level (*P *= .059). Data presented as mean ± SEM; n = 11. ACC: Anterior Cingulate Cortex; NAc: Nucleus Accumbens; NOP: Nociceptin/orphanin FQ peptide; PRT: Probabilistic Reward Task.

Contrary to our hypotheses, the treatment × block ANOVA revealed no effects involving treatment (*P* > .99) (Figure 1A).

### Feedback-Locked ERP

A significant treatment × reward feedback × stimulus type interaction emerged for the ACC (F_[1,10]_ = 10.38; *P* = .009; NAc: F_[1,10]_ = 3.77, *P* = .084). For the ACC, follow-up treatment × reward feedback ANOVAs for each stimulus revealed a treatment × reward feedback interaction for the lean but not rich stimulus (lean: F_[1,10]_ = 8.18, *P* = .017; rich: *P* > .93). Post-hoc tests showed that the interaction was driven by a trend (*P* = .059) toward more positive deflection to nonrewarded lean trials for J-113397 than vehicle (Figure 1B and C).

### Feedback-Locked 1-5 Hz (Delta) Frequency Band

**Figure 2.**
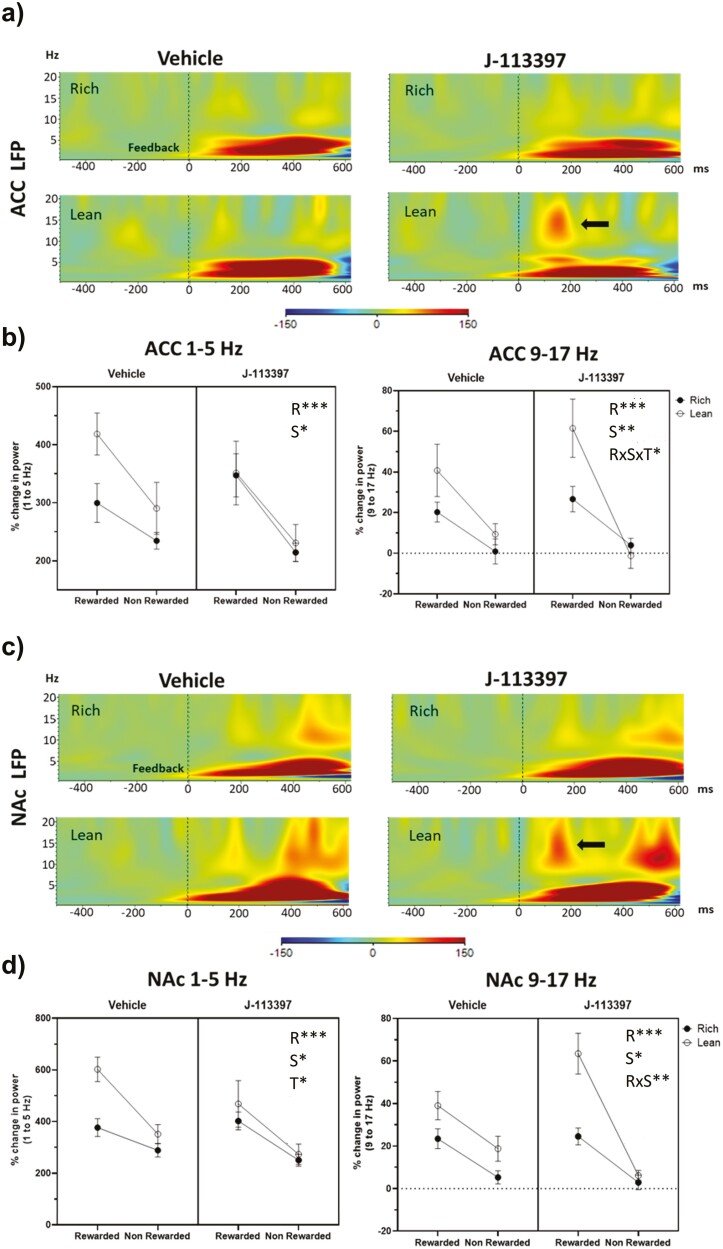
Effect of the NOP antagonist J-113397 on reward-related changes in delta (1–5 Hz) and alpha-beta (9–17 Hz) power. (A) Grand average of feedback-locked wavelet-decomposed time-frequency spectra for correct rich/lean trials presented as the difference between rewarded minus nonrewarded trials for the ACC. The black arrow highlights the increase in 9 to 17-Hz power for J-113397 lean trials. (B) Feedback-locked mean delta power (1–5 Hz) at 200–600 milliseconds and mean 9 to 17-Hz power at 100–200 milliseconds in ACC for correct rewarded and nonrewarded trials separated by stimulus type. (C) and (D) show the analogous figures for the NAc LFP channel as presented in (A) and (B) for the ACC. The main effect of J-113397 on delta power with overall lower delta power for J-113397 relative to vehicle was found. Similarly, a treatment × reward feedback × stimulus type interaction for ACC 9 to 17-Hz power emerged. Follow-up analyses clarified that this difference was driven by a higher 9 to 17-Hz power for J-113397 relative to vehicle exclusively for lean rewarded trials (*P* = .021). Main effects and interaction are presented with letters R (reward/nonreward feedback), S (stimulus), R × S (interaction), and T (treatment) with asterisks according to their statistical significance. Data presented as mean ± SEM; n = 11. ACC: Anterior Cingulate Cortex; LFP: Local Field Potentials; NAc: Nucleus Accumbens; NOP: Nociceptin/orphanin FQ peptide.

Compared with nonrewarded, rewarded trials elicited overall significantly higher delta power (200–600 milliseconds) in ACC and NAc (reward feedback: ACC: F_[1,10]_ = 62.68; *P* < .0001; NAc: F_[1,10]_  = 24.12; *P* = .001) (Figure 2A–D). Similarly, a main effect of stimulus type emerged in ACC and NAc (ACC: F_[1,10]_ = 4.96; *P* = .05; NAc: F_[1,10]_ = 10.06; *P* = .010) as the lean stimulus (which, per design, is associated with the largest reward prediction error) showed overall higher delta power. Interestingly, a main effect of J-113397 was found exclusively in NAc (F_[1,10]_ =  7.94; *P* = .018) due to lower delta power for J-113397 relative to vehicle (Figure 2C and D).

### Feedback-Locked 9-17 Hz Frequency Band

Reward feedback elicited significantly higher 9 to 17-Hz power (100–200 milliseconds) in ACC and NAc relative to nonrewarded trials (reward feedback: ACC: F_[1,10]_ = 69.60; *P* < .0001; NAc: F_[1,10]_ = 43.38; *P* < .0001) (Figure 2A–D). A main effect of stimulus type for both electrodes was also found (ACC: F_[1,10]_ = 12.46; *P* = .005; NAc: F_[1,10]_ = 5.31; *P* = .044). Critically, for the ACC, this effect was qualified by a significant treatment × reward feedback × stimulus type interaction (F_[1,10]_ = 5.52, *P* = .041). Follow-up analyses clarified that the treatment × reward interaction was significant for the lean (F_[1,10]_  = 7.87, *P* = .019) but not rich stimulus (*P* > .65). This effect was driven by a higher 9 to 17-Hz spectral power for J-113397 relative to vehicle for rewarded lean trials (*P* = .021) (Figure 2C and D).

## DISCUSSION

Our findings provide initial evidence that a single administration of a NOP antagonist modulated electrophysiological markers of reward learning without affecting behavior. While these preliminary findings suggest that electrophysiological markers might be especially sensitive in detecting effects of NOP antagonism, replications in larger samples are warranted. Notably, in addition to a general reduction in delta power in NAc (irrespective of stimulus type and reward delivery) by J-113397, the most specific drug effect was observed for the feedback-locked 9 to 17-Hz frequency band, which was significantly larger in ACC for J-113397 relative to vehicle in response to rewarded lean trials. These findings are intriguing in light of human findings showing that beta power is potentiated by delivery of unexpected (low probability) rewards (HajiHosseini et al., 2012; Marco-Pallares et al., 2015), which in the PRT correspond to rewarded lean trials. Because unexpected rewards have been linked to dopaminergic signaling, these findings raise the possibility that NOP antagonism might potentiate dopaminergic signaling to salient cues. Future dose-response studies are warranted to evaluate this speculation and evaluate the promise of NOP antagonism to reverse anhedonic phenotypes.

## Data Availability

The data underlying this article will be shared on reasonable request to the corresponding author.

## Interest Statement

Over the past 3 years, Dr Pizzagalli has received consulting fees from Albright Stonebridge Group, Boehringer Ingelheim, Compass Pathways, Engrail Therapeutics, Karla Therapeutics, Neumora Therapeutics (formerly BlackThorn Therapeutics), Neurocrine Biosciences, Neuroscience Software, Otsuka, Sage Therapeutics, Sunovion, and Takeda; he has received honoraria from the Psychonomic Society and American Psychological Association (for editorial work) and from Alkermes; he has received research funding from the Brain and Behavior Research Foundation, Dana Foundation, Wellcome Leap, Millennium Pharmaceuticals, and NIMH; he has received stock options from Compass Pathways, Engrail Therapeutics, Neumora Therapeutics, and Neuroscience Software; he has a financial interest in Neumora Therapeutics, which has licensed the copyright to the human version of the probabilistic reward task through Harvard University. No funding from these entities was used to support the current work, and all views expressed are solely those of the authors. All other authors have no conflicts of interest or relevant disclosures.
